# Does external pneumatic compression treatment between bouts of overreaching resistance training sessions exert differential effects on molecular signaling and performance-related variables compared to passive recovery? An exploratory study

**DOI:** 10.1371/journal.pone.0180429

**Published:** 2017-06-29

**Authors:** Cody T. Haun, Michael D. Roberts, Matthew A. Romero, Shelby C. Osburn, Christopher B. Mobley, Richard G. Anderson, Michael D. Goodlett, David D. Pascoe, Jeffrey S. Martin

**Affiliations:** 1 School of Kinesiology, Auburn University, Auburn, Alabama, United States of America; 2 Department of Cell Biology and Physiology, Edward Via College of Osteopathic Medicine – Auburn Campus, Auburn, Alabama, United States of America; 3 Athletics Department, Auburn University, Auburn, Alabama, United States of America; University of Alabama at Birmingham, UNITED STATES

## Abstract

**Purpose:**

We sought to compare the effects of external pneumatic compression (EPC) and sham when used concurrently with resistance training on performance-related outcomes and molecular measures related to recovery.

**Methods:**

Twenty (N = 20) resistance-trained male participants (aged 21.6±2.4 years) were randomized to balanced sham or EPC intervention groups. The protocol consisted of 3 consecutive days of heavy, voluminous back squat exercise followed by EPC/sham treatment (Days2-4) and 3 consecutive days of recovery (Days5-7) with EPC/sham only on Days5-6. On Day1 (PRE), and Days3-7, venipuncture, flexibility and pressure-to-pain threshold (PPT) measures were performed. *Vastsus lateralis* muscle tissue was biopsied at PRE, 1-h post-EPC/sham treatment on Day2 (POST1) and 24-h post-EPC/sham treatment on Day7 (POST2). Isokinetic peak torque was assessed at PRE and POST2.

**Results:**

Peak isokinetic strength did not change from PRE to POST2 in either group. The PPT was significantly lower on Days3-6 with sham, indicating greater muscle soreness, though this was largely abolished in the EPC group. A significant decrease in flexibility with sham was observed on Day3 (+16.2±4.6% knee joint angle; *P*<0.01) whereas there was no change with EPC (+2.8±3.8%; *P*>0.01). *Vastus lateralis* poly-ubiquitinated proteins significantly increased at the POST2 time point relative to PRE with sham (+66.6±24.6%; *P*<0.025) and were significantly greater (P<0.025) than those observed with EPC at the same time point (-18.6±8.5%). 4-hydroxynonenal values were significantly lower at POST2 relative to PRE with EPC (-16.2±5.6%; *P*<0.025) and were significantly lower (P<0.025) than those observed with sham at the same time point (+11.8±5.9%).

**Conclusion:**

EPC mitigated a reduction in flexibility and PPT that occurred with sham. Moreover, EPC reduced select skeletal muscle oxidative stress and proteolysis markers during recovery from heavy resistance exercise.

## Introduction

Lower limb compression has been utilized in order to enhance skeletal muscle adaptations and/or recovery from high-intensity exercise (i.e. recovery-adaptation). For instance, lower-limb compression garments have been shown to reduce serum creatine kinase (CK) levels, 1.5 and 3.5 days following rugby participation compared to a passive recovery method [1]. Likewise, compression garments have been shown to lower post-eccentric exercise CK levels, preserve range of motion in the elbow flexors, reduce perceived soreness and swelling, and promote force production recovery [2]. However, other investigations have refuted the use of static compression garments on improving sports performance, post-exercise lactate, and/or post-exercise markers of muscle damage and/or recovery [3, 4].

Commercial external pneumatic compression (EPC) devices differ from static compression in that they utilize whole-leg sleeves that operate by inflating and deflating a series of zones using modest inflation pressures (~20–100 mm Hg). While it has been posited that EPC can exert localized vasoactive effects [5] and improve post-exercise lactate clearance [6], EPC devices are also putatively analogous to a lower-limb massage which has been shown to decrease exercise-induced inflammatory signaling [7]. Interestingly, investigators have reported that a single 15 min EPC treatment acutely improves flexibility [8] and increases the pressure-to-pain threshold (PPT) in the lower limbs following resistance training [9]. We have also published a series of studies examining how moderate pressure EPC affects transcriptomic and signaling responses in skeletal muscle after one acute treatment [10, 11] and after seven consecutive days of treatment [12] in healthy persons. We have reported that a 1 h treatment of EPC increases intramuscular phosphorylated ribosomal protein s6 levels by 31% at 1 h post-treatment, while also increasing interleukin-10 (IL-10) and superoxide dismutase 2 (SOD2) mRNA 1 h post-treatment [10, 11]. Moreover, we have previously reported that seven consecutive days of moderate-pressure EPC increases the mRNA expression of genes related to skeletal muscle hypertrophy (i.e., MHY2, IGFBP5, MYOM1) and oxidative stress resilience (i.e., CAT, SUOX) [12].

Despite the aforementioned studies suggesting that EPC can potentially modulate post-exercise inflammation and/or skeletal muscle signaling events, little mechanistic research has examined how utilizing EPC as an adjuvant to heavy resistance exercise affects functional recovery and the intramuscular signaling phenomena. We have previously utilized a heavy, voluminous, three consecutive day barbell back squat protocol and demonstrated significant increases in serum markers of muscle damage (i.e. myoglobin) and perceived soreness (i.e. self-reported analog scale) as well as a decrease in strength/performance (i.e. isometric peak knee extensor torque) 48-h following the last back squat session in a control group.[13] Thus, we chose to use the same protocol herein with and without EPC treatment, to examine if: a) EPC treatment after one bout of heavy squat exercise differentially affected mRNA and protein expression patterns related to inflammation, muscle hypertrophy, proteolysis and/or oxidative stress compared to sham, and b) if three consecutive days of EPC treatment immediately after heavy, voluminous barbell back squats and an additional two days of treatment alone affected the aforementioned molecular markers as well as soreness and performance-related variables in comparison to sham.

## Methods

### Participant characteristics

Prior to initiating this study, the protocol was reviewed and approved by the Auburn University Institutional Review Ethics Committee, and was in compliance with the Helsinki Declaration. Apparently healthy males (N = 20) volunteered to take part in this investigation. Subjects gave written consent and completed the Physical Activity Readiness Questionnaire as well as a health history questionnaire to detect potential risk factors that might be aggravated by strenuous physical activity. All participants were considered resistance-trained, participating in ≥ 3 days per week of full-body resistance exercise for at least 3 months.

### Experimental protocol

**Fig 1:**
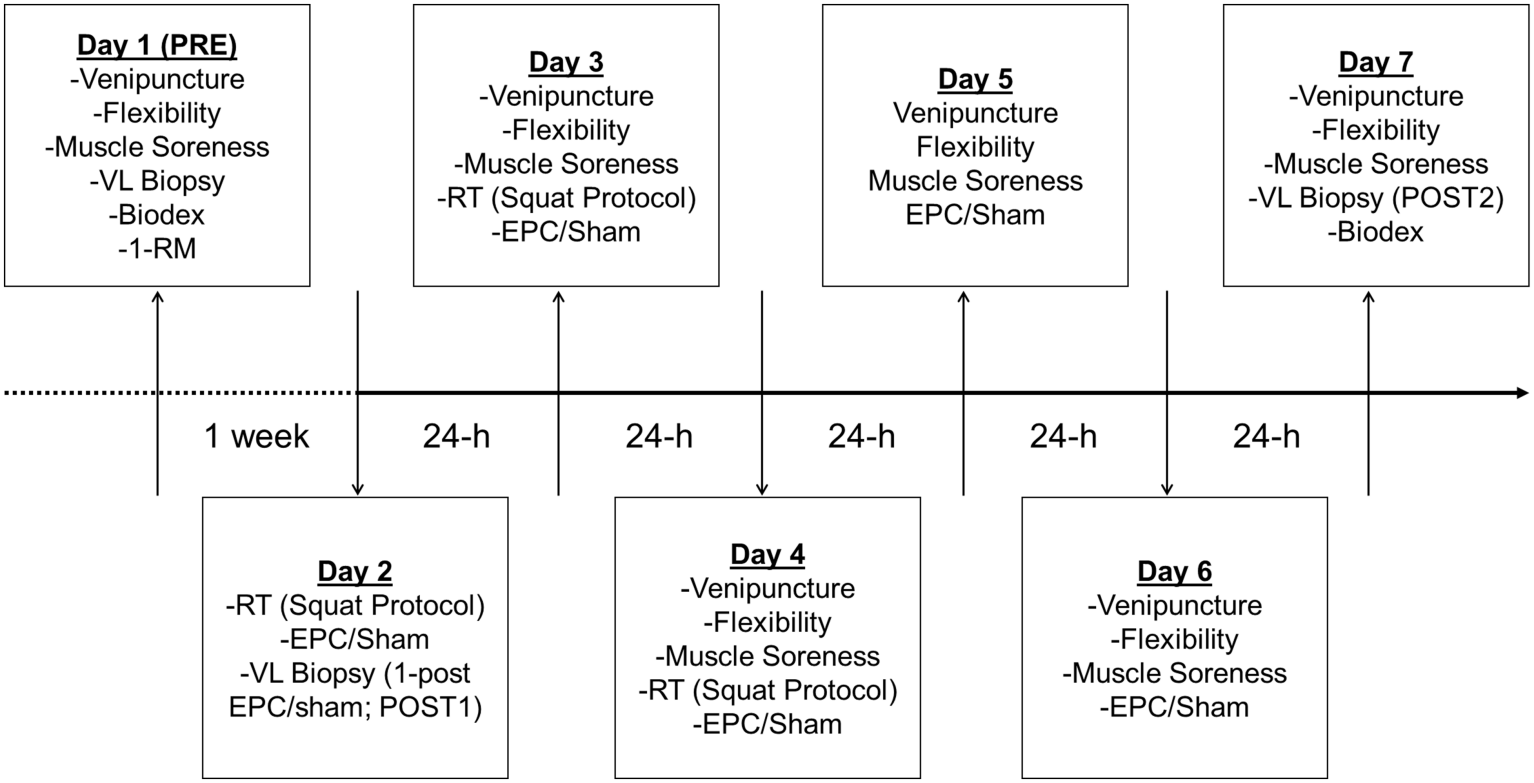
Time and events associated with the protocol utilized herein. On what is termed as Day 1 (i.e., PRE), participants’ baseline right knee range of motion (i.e., flexibility), pressure-to-pain threshold in the right *vastus lateralis* (i.e., muscle soreness), and peak isokinetic right knee extensor torque (Biodex) values were established. In addition, venipuncture and a *vastus lateralis* biopsy of the left leg was performed for subsequent analyses. Finally, 1-RM back squat values were established for determination of training loads. Participants then reported to the laboratory exactly 1 week later (i.e. Day 2; POST1) and performed 10 sets of 5 reps of the back squat at 80% of their established 1-RM. This was immediately followed by randomization to either the external pneumatic compression (EPC) or sham treatment group. According to group assignment, immediately following resistance training, participants were treated for 1 h. 1 h following treatment a second biopsy of the left *vastus lateralis* was performed. On the next 2 consecutive days (Day 3 and 4) participants completed the same resistance training followed by respective treatment protocol. On these days, venipuncture and muscle soreness and flexibility assessments were performed prior to resistance training. On the next 2 consecutive days (Days 5–6) venipuncture and the muscle soreness and flexibility assessments were performed and were followed by treatment according to group assignment (no resistance training was performed). Finally, on Day 7 (i.e., POST2) participants reported to the lab for venipuncture and the muscle soreness and flexibility assessments. Thereafter, a third biopsy of the left *vastus lateralis* was performed and peak isokinetic right knee extensor torque was measured.

Fig 1 provides an outline of the experimental protocol. The protocol is described more in-depth below and the experimental procedures used in the protocol are described thereafter.

#### Day 1 (pre-testing [PRE])

Participants were requested to report to the laboratory following a 4 h fast to control for any metabolic influence on study outcomes. In addition, participants were asked to forgo any strenuous activity for at least 48 h prior to arrival in order to minimize any residual markers of muscle damage. Baseline venous blood samples were then collected into a 5 mL serum separator tube and a 3 mL EDTA tube (BD Vacutainer, Franklin Lakes, NJ, USA) for subsequent analysis of serum and plasma markers, respectively, described below. Participants were then instructed to lay in a supine position on a treatment table whereby a baseline (PRE) percutaneous skeletal muscle biopsy was obtained from the left *vastus lateralis* midway between the patella and iliac crest using a 5 gauge needle with suction and sterile laboratory procedures. Briefly, 1.5 mL of 1% Lidocaine was injected subcutaneously above the skeletal muscle fascia prior to making a small pilot incision for needle intrusion. The biopsy needle was then inserted at a depth just beyond the fascia and approximately 100–150 mg of skeletal muscle was removed using a double-chop method and applied suction. Extracted tissue was immediately blotted of visible blood using sterile gauze pads and had all visible fat and connective tissue removed. Thereafter, approximately 50 mg tissue was immediately placed in a 1.7 mL polypropylene tube containing 500 μL of cell lysis buffer (Cell Signaling, Danvers, MA, USA) with pre-added protease and phosphatase inhibitors and processed for protein analyses as described below. Additionally, 10–20 mg of muscle was placed in a 1.7 mL polypropylene tube containing 500 μL of Ribozol (Ameresco, OH, USA) for mRNA analyses as described below, and the remaining tissue was snap-frozen in liquid nitrogen and subsequently stored at -80°C.

Flexibility was assessed by measuring knee range of motion during a modified kneeling lunge similar to the methods described by MacDonald et al [14]. Briefly, participants were positioned in the modified lunge position (upright and erect torso, left knee in line with left ankle perpendicular to floor, right knee in contact with floor behind the torso to the point of stretch-induced discomfort in the right hip) and the right hip angle was visually confirmed for positioning on all days of flexibility assessment. Thereafter, the subject’s right knee was passively flexed by an investigator until the participant verbally noted the point of discomfort. The knee angle, in degrees, at this point of stretch was recorded with a goniometer using the lateral malleolus and lateral epicondyle along with the center of the *vastus lateralis* as landmarks.

Muscle soreness was measured by applying focal pressure to proximal, medial, and distal targeted areas of the right *vastus lateralis* using an instrumented algometer (Force Ten FDX, Wagner Instruments, Greenwich, CT, USA). The vastus lateralis was visually divided into the three sections (proximal, medial and distal) with markings made for pressure transducer application placed at the center of each section. Markings were made with permanent marker and remained visible throughout the duration of the study for each subject with occasional “touch-up” of the marks when fading was noted. The algometer pressure transducer was applied at a rate of approximately 5 N/s at each site until the subject indicated the point at which the pressure became painful. The point at which the pressure became painful (audibly indicated by participants) was termed the PPT and the value in N was recorded. The PPT was measured sequentially in cycles from the proximal to medial to distal site three times for triplicate measures. The average of the triplicate measures at each site was calculated as the respective PPT. Notably, the digital display of the algometer indicating force of application was blinded to the participants.

To assess strength/performance in the lower limbs, participants were then seated on the System 4 Pro Biodex isokinetic dynamometer (Biodex Medical Inc., New York, NY, USA). Maximal knee extensor isokinetic torque of the right leg was measured across 5 repetitions at 60°/s with a brief rest period (~1–2 min) between bouts. We chose an isokinetic speed of 60°/s given the moderately strong correlation between isokinetic knee extension and functional performance previously reported in the literature [15]. Moreover, although assessment of strength/performance using a more dynamic movement and/or similarity to the back squat was preferable, we chose to use isokinetic testing to 1) perform unilateral testing in a limb that had not been subjected to percutaneous skeletal muscle biopsies and 2) to better isolate the strength/performance to an area at which EPC was applied (e.g. reducing the influence of the back extensors) and from which molecular analyses would be derived (i.e. *vastus lateralis*).

A 5–10 min recovery period was allowed following isometric/isokinetic dynamometry. Following this recovery period, participants began a one repetition maximum (1RM) back squat protocol. The first set of back squats occurred with the bar only (20 kg) for 5 repetitions. Participants were allowed a 2–3 min recovery period if desired (minimum of 1 min.) and then performed 5 repetitions at ~50% of their putative 1RM. Participants were allowed a 2–3 min recovery period and then performed 2 repetitions at ~80% of their putative 1RM. Thereafter, participants performed 1 rep whereby ~2–5 kg were added until they were unable to achieve a successful lift or consensus between investigator and subject was reached regarding the 1RM value. To ensure that proper depth on each back squat repetition was accomplished, participants were asked to make contact with a box that was behind them without sitting on it entirely. The box was set at a height where the participant’s femur was approximately parallel to the ground during the bottom-eccentric portion of the back squat. Following 1RM back squat testing, participants were scheduled for subsequent experimental visits. In addition, they were asked to not perform any strenuous physical activity for at least 48 h prior to their day 2 visit described below.

#### Day 2 (resistance training & treatment bout 1)

**Fig 2:**
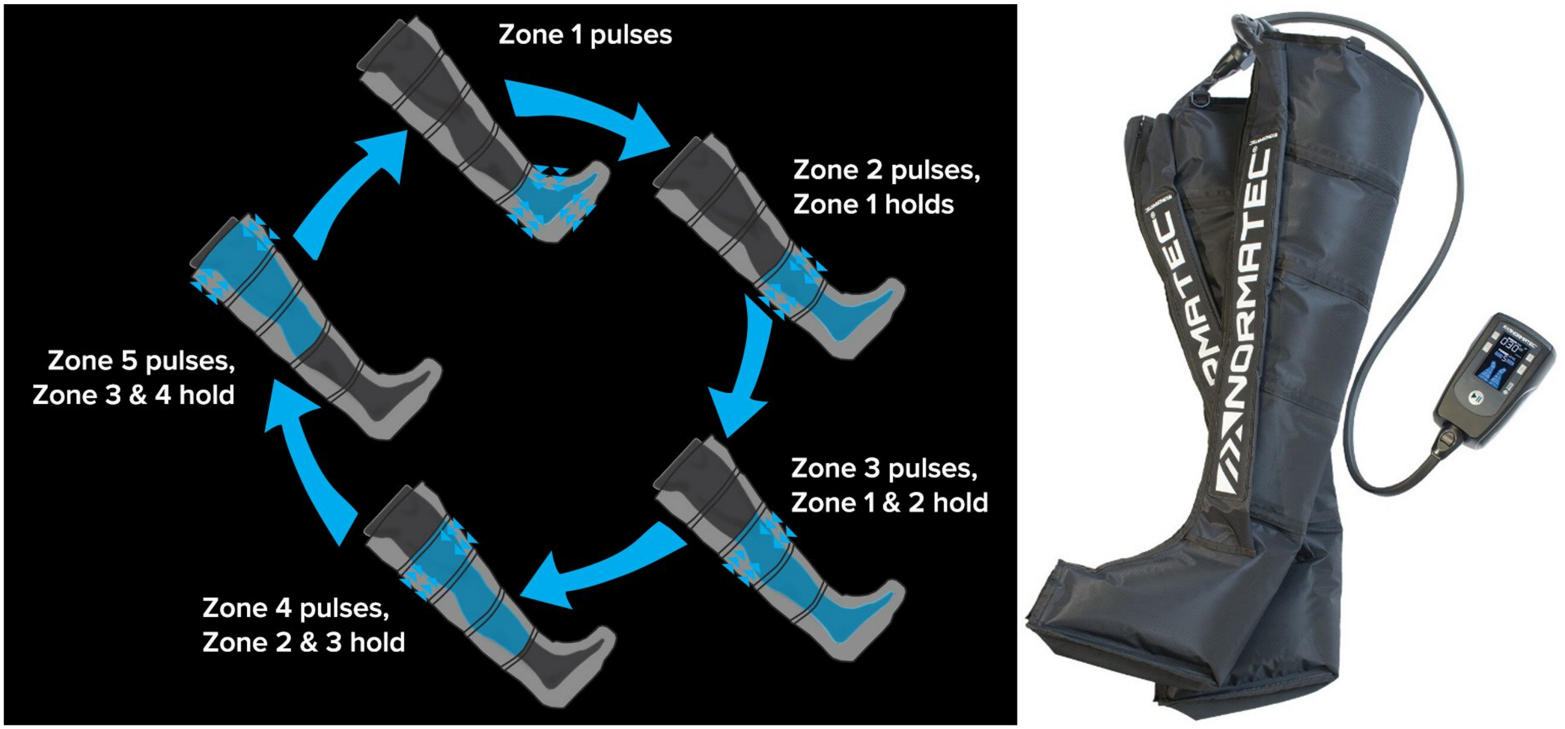
Representative image of the external pneumatic compression (EPC) device utilized herein. Pictured is the external pneumatic compression (EPC) device and leg sleeves (right) and an illustration of the peristaltic pulse massage pattern (left).

Approximately one week following Day 1 (PRE), participants were requested to report to the laboratory 4 h fasted at approximately the same time of day as the Day 1 testing. Participants performed a back-squat warm up protocol of 10 repetitions with the barbell (i.e., 20 kg), and 10 repetitions at ~50% of their 1RM. Following the warm-up sets, participants performed 10 sets of 5 repetitions at 80% of their back squat 1RM with a 2–3 min recovery period between each set. If participants were unable to complete the repetitions, a 5% reduction in weight was employed. Lifting volumes for the exercise bouts were recorded and included dropped weights. Immediately following completion of the exercise bout, participants were randomly assigned to either a 1 h treatment with EPC (NormaTec, Newton, MA, USA) or no compression (sham) whereby the participants had the leg sleeve on but not inflated for 1 h. The EPC device consists of two separate “leg sleeves” which contain five circumferential inflatable chambers (arranged linearly along the limb) encompassing the leg from the feet to the hip/groin. The “leg sleeves” are connected to an automated pneumatic pump at which target inflation pressures for each zone and the duty cycle can be controlled. However, the unit is commercially marketed with pre-programmed defaults for the duty cycle and recommended inflation pressure settings. In this study we chose to use an inflation protocol consisting of target inflation pressures of ~70 mm Hg for each chamber and a duty cycle that included 30 s of compression in each zone followed by a 30 second rest period during which all zones are deflated. We have previously investigated and described this protocol and system [6, 16] and a simple illustration of the compression pattern delivered by the EPC system is presented in Fig 2. 1 h following the conclusion of EPC or sham treatments, participants donated a second muscle biopsy from the left *vastus lateralis* (termed POST1) using techniques and processing procedures described above.

#### Days 3–4 (resistance training & treatment bouts 2 and 3)

For Days 3–4, 24 h following the previous visit, participants reported to the laboratory 4 h fasted. Venous blood sampling and assessment of flexibility of the right leg and the PPT in the right *vastus lateralis* was performed via methods described above. Participants then performed the 10 sets of 5 repetitions at 80% of their back squat 1RM described during day 2 above. Thereafter, participants were again treated for 1 h with either EPC or sham.

#### Days 5–6 (treatment only bouts 4 and 5)

For days 5–6 (which occurred after the three consecutive resistance exercise bouts), participants reported the laboratory 4 h fasted. Venous blood sampling and assessment of flexibility of the right leg and the PPT in the right *vastus lateralis* was performed via methods described above. Thereafter, participants were again treated for 1 h with either EPC or sham.

#### Day 7 (post-testing)

On the post-testing day, participants reported to the laboratory 4 h fasted and venous blood sampling and assessment of flexibility of the right leg and the PPT in the right *vastus lateralis* was performed via methods described above. Next, a final biopsy from the left *vastus lateralis* was obtained, and tissue was processed for protein and RNA analyses as described above. Notably, for the three *vastus lateralis* biopsies, each one was obtained slightly proximal to the previous one(s) as this sampling sequence may prevent variability due to inflammatory signaling that can occur with multiple biopsy sampling [17]. Finally, post-study maximal knee extensor isokinetic torque of the right leg was again measured across 5 repetitions at 60°/s and 120°/s, with a brief rest period (~1–2 min) between bouts.

#### Other notes

Participants were asked to maintain their habitual dietary and sleep habits, not use any “recovery” aids (e.g. ice, topical analgesics, etc.) and to abstain from taking aspirin or NSAIDS throughout the study. Moreover, participants reported to the laboratory for testing and resistance training during the same time of day (± 1 hr) throughout.

### Experimental procedures

#### Serum and plasma analyses

On the days of blood collection, serum and plasma tubes were centrifuged at 3,500 x g for 5 min at room temperature. Aliquots were then placed in 1.7 mL microcentrifuge tubes and stored at -80°C until batch-processing. Human ELISAs were used to determine serum concentrations of interleukin-6 (IL-6) (Cayman Chemical, Ann Arbor, MI) and plasma levels of C-reactive protein (CRP) (Cayman Chemical). An activity assay was used to determine serum levels of CK (Bioo Scientific, Austin, TX). All kits were performed according to manufacturer’s instructions and plates were read using a 96-well spectrophotometer (BioTek, Winooski, VT).

#### RNA expression analyses

Immediately following muscle extraction, samples were homogenized in 500 μL of Ribozol (Ameresco) and stored at -80°C for batch processing. During batch processing, total RNA isolation occurred according to manufacturer’s instructions. RNA concentrations were subsequently assessed using a NanoDrop Lite (Thermo Scientific, Waltham, MA, USA) prior to cDNA synthesis for mRNA analyses. cDNA synthesis was reverse transcribed from 1,000 ng of total RNA for real time PCR analyses using a commercial cDNA synthesis kit (Quanta Biosciences, Gaithersburg, MD, USA). Real-time PCR was performed using SYBR-green-based methods with gene-specific primers designed using an online primer designer tool (Primer3Plus, Cambridge, MA, USA). Fold-change values within each subject from the PRE biopsy were performed using the Livak method [18], where -ΔΔCT = (post-EPC gene of interest–post-EPC geometric mean of GAPDH)–(pre-EPC gene of interest–pre-EPC GAPDH). Following the PCR reaction for each gene, melt curve analyses were performed to ensure that one PCR product was amplified per reaction. A list of targeted markers and PCR primers is listed in Table 1.

**Table 1:** Primer sequences for real-time PCR.

Gene	Forward primer (5’ →3’)	Reverse primer (5’ → 3’)
*Hypertrophy and proteolysis*		
MGF	CGAAGTCTCAGAGAAGGAAAGG	ACAGGTAACTCGTGCAGAGC
MSTN	GACCAGGAGAAGATGGGCTGAATCCGTT	CTCATCACAGTCAAGACCAAAATCCCTT
MuRF-1	GCCTTCTTCGCCTTCTCC	AGCTCATACAGACTCAGTTCC
Atrogin-1	ATGTGCGTGTATCGGATGG	AAGGCAGGTCAGTGAAGC
*Ribosome Biogenesis*		
c-Myc	ACAACCGAAAATGCACCAGC	CGTTGTGTGTTCGCCTCTTG
TAF1B	TCTTGAAAGTGGAGCGGAGT	GTGTCTGTGGCATGGTCATC
45S pre-rRNA	GAACGGTGGTGTGTCGTT	GCGTCTCGTCTCGTCTCACT
28S	GCGTTGGATTGTTCACCCAC	ACCTGTCTCACGACGGTCTA
Exportin-5	TGCCCTTCCATGAATGACCC	CTGAGTGGACCTTGAGGCTG
*Inflammation*		
IL-6	AGGAGACTTGCCTGGTGAAA	CAGGGGTGGTTATTGCATCT
IL-10	GAAGAGAAACCAGGGAGCCC	ATCCCTCCGAGACACTGGAA
IL-1β	AAGGCGGCCAGGATATAACT	CCCTAGGGATTGAGTCCACA
MCP-1	TCCCAAAGAAGCTGTGATCTTCA	CAGATTCTTGGGTGGAGTGA
TNF-α	TCCTTCAGACACCCTCAACC	AGGCCCCAGTTTGAATTCTT
*Satellite cell-related genes*		
MyoD	AAGCGCCATCTCTTGAGGTA	GCGAGAAACGTGAACCTAGC
Myogenin	GCCAGACTATCCCCTTCCTC	GAGGCCGCGTTATGATAAAA
Pax7	AGGGCCTCCTGCTTGTTTAT	GGTTTTGCCCAACTCAGTGT
MHCemb	GAGGAGGCTGATGAACAAGC	TCCTGCTGGAGGTGAAGTCT
*Housekeeping genes*		
Fbl	CCCACACCTTCCTGCGTAAT	GCTGAGGCTGTGGAGTCAAT
GAPDH	AACCTGCCAAATATGATGAC	TCATACCAGGAAATGAGCTT

All primers were designed using PrimerPlus3 (Cambridge, MA, USA) and BLASTed against other potential mRNA targets using the online NCBI Nucleotide database (Bethesda, MD). MGF, mechano-growth factor; mstn, myostatin; murf-1, muscle RING finger 1; TAF1B, TATA-box protein associated factor, RNA polymerase 1 subunit B; 45S pre-rRNA, 45S pre-ribosomal 5; IL, interleukin; MCP-1, monocyte chemoattractant protein-1; TNF-α, tumor necrosis factor α; MyoD, myogenic differentiation 1; Pax7, paired box 7; MHCemb, myosin heavy chain embryonic; fbl, fibrillarin; GAPDH, glyceraldehyde 3-phosphate dehydrogenase.

#### Western blotting analyses

Immediately following muscle extraction, samples were homogenized using a tight-fitting micropestle in cell lysis buffer described above, insoluble proteins were removed with centrifugation at 500 x g for 5-min at 4°C, and supernatants containing muscle tissue homogenate were collected and stored at -80°C. After all participants finished the study, muscle tissue homogenates were batch-assayed for total protein content using a BCA Protein Assay Kit (Thermo Scientific, Waltham, MA, USA).

Cell lysis homogenates obtained from above were prepared for Western blotting using 4x Laemmli buffer at 1 μg/μL. Thereafter, 20 μL of prepped samples were loaded onto 12% SDS-polyacrylamide gels (BioRad, Hercules, CA, USA) and subjected to electrophoresis (180 V @ 60-min) using pre-made 1x SDS-PAGE running buffer (Ameresco). Proteins were then transferred to polyvinylidene difluoride membranes (BioRad), Ponceau stained and imaged to ensure equal protein loading between lanes. Thereafter, membranes were blocked for 1 h at room temperature with 5% nonfat milk powder. Rabbit anti-IGF-1 IgG (1:1,000; Hybridoma Bank, Iowa City, IA, USA), rabbit anti-phosphorylated 4EBP1 (Thr37/46) (1:1,000; Cell Signaling), rabbit anti-phosphorylated p70s6k (Thr389) (1:1,000; Cell Signaling), rabbit anti-phosphorylated rps6 (Ser235/236) (1:1,000; Cell Signaling), rabbit anti-phosphorylated IkBα (Ser32) (1:1,000; Cell Signaling), rabbit anti-pan IkBα (1:1,000; Cell Signaling), rabbit anti-phosphorylated p65/NF-κB (Ser536) (1:1,000; Cell Signaling), rabbit anti-pan p65/NF-κB (1:1,000; Cell Signaling), rabbit anti-20S core (1:1,000; Millipore), rabbit anti-ubiquitin (1:1,000; Cell Signaling), rabbit anti-4HNE (1:1,000; Abcam, Cambridge, MA, USA), and rabbit anti-catalase (1:1,000; GeneTex, Irvine, CA, USA) were incubated with membranes overnight at 4°C in 5% bovine serum albumin (BSA), and the following day membranes were incubated with horseradish peroxidase-conjugated anti-rabbit IgG (1:2,000, Cell Signaling) at room temperature for 1 h prior to membrane development. Membrane development was performed using an enhanced chemiluminescent reagent (Luminata Forte HRP substrate; Millipore, Billerica, MA USA), and band densitometry was performed through the use of a gel documentation system and associated densitometry software (UVP, Upland, CA, USA). Of note, the densitometry values for all protein targets were normalized to Ponceau densities, and these values were normalized to PRE values in order to obtain fold-change values from 1.00. Phosphorylated rps6 levels were highly erratic (i.e., either very highly expressed or not detectable) and, thus, are not presented. Furthermore, phosphorylated p65/NF-κB and IkBα were only detected in ~1% of the samples and, thus, pan levels of these targets were analyzed and presented.

### Statistics

Unless otherwise stated, all variables are presented in figures and tables as means ± standard error values and an alpha (α) level of p≤0.05 was used to detect between- or within-group differences. All statistics were performed using SPSS v22.0 (Chicago, IL, USA). Independent t-tests were performed for pre-study age, height, weight, BMI, and 1RM squat strength. Isokinetic knee extensor torque was analyzed using 2x2 (group*time) mixed factorial ANOVAs and total lifting volume and mRNA data was analyzed using 2x3 (group*time) mixed factorial ANOVAs. Serum analyte concentrations, flexibility and muscle soreness measurements were analyzed using 2x5 (group*time) repeated measures MANOVAs with PRE values included in the model as a covariate. If a significant main effect of the within-subjects factor of time or the between subjects factor of group was observed for a dependent variable, subsequent pairwise comparisons with Bonferroni adjustments were applied (α/[number of comparisons-1]). If a significant group*time α-value was observed for a dependent variable, independent t-tests at each time point were performed at each time point with Bonferroni correction for multiple comparisons (α/[number of time points-1]). Effect sizes were also investigated using Cohen’s guidelines of moderate and large effects with ES values of 0.50 and 0.80, respectively [19]. Effect sizes were calculated as the ratio of the respective mean difference from PRE in the EPC and sham groups at a given time point to the pooled standard deviation of the differences. It should be finally noted that, for all dependent variables analyzed with mixed factorial ANOVAs, Mauchly’s tests of sphericity were performed to assure that the variances of all groups are equal and that the data was normally distributed. In the event that sphericity was not met, the Huynh-Feldt correction was applied to hypothesis testing.

## Results

### Participant characteristics

Participants enrolled in the sham group were, on average, 22.5 ± 0.8 years old with a mean BMI of 24.7 ± 0.9 kg/m^2^ (height: 1.82 ± 0.24 m, body mass: 82.2 ± 3.6 kg). In the EPC group, participants were, on average, 20.8 ± 0.6 years old with a mean BMI of 25.6 ± 1.6 kg/m^2^ (height: 1.77 ± 0.39 m, body mass: 78.9 ± 3.2 kg). Mean back squat 1RM at baseline was 128.6 ± 9.5 kg and 137.1 ± 12.8 kg in the sham and EPC groups, respectively. Importantly, there were no between-group differences in height (*P* = 0.08), body mass (*P* = 0.56), body mass index (*P* = 0.63), age (*P* = 0.09), or back squat 1RM (*P* = 0.52).

### Effects of EPC on pressure-to-pain threshold (PPT) and flexibility

**Fig 3:**
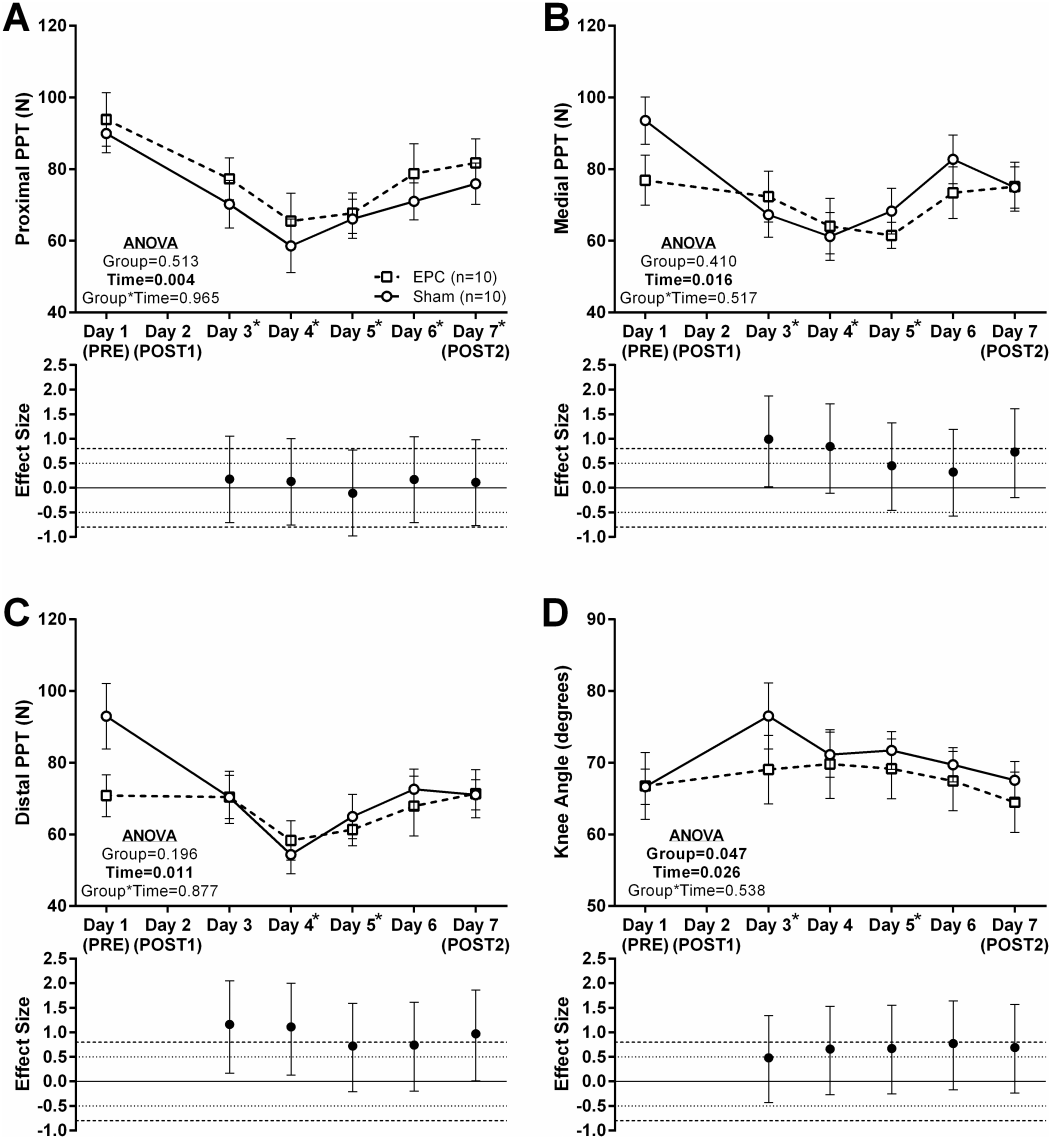
Changes in right *vastus lateralis* pressure-to-pain threshold and flexibility about the right knee. Pressure-to-pain threshold (PPT) along the right *vastus lateralis* at a proximal, medial and distal site is presented in Panels A-C. The PPT threshold was assessed via muscle algometry with pressure applied at a rate of ~5 N/s until the subject verbally indicated the pressure had turned into pain. Cyclical PPT measurements were made in triplicate. Flexibility assessed via right knee range of motion in a modified lunge position is presented in Panel D. For all panels data are presented as mean ± standard error and effect sizes for change from PRE are presented as d ± 95% confidence interval. Effect sizes were calculated as mean change from PRE in the EPC group less mean change from PRE in the sham group divided by the pooled standard deviation for each respective time point. Repeated measures MANOVA was performed with PRE values as a covariate and an α ≤ 0.05 required for statistical significance. Main effects of time and/or group are presented as emphasized p-values. *Post-hoc* testing for a main effect of time was performed using using Student’s paired t-tests and an α ≤ 0.01 was required for statistical significance. *, time point (collapsed across groups) significantly different from PRE.

With regards to the PPT, a main effect of time was observed for the proximal, medial and distal sites of measurement (Fig 3A–3C). However, neither a main effect of group nor a group*time interaction was observed for any of the sites. At the proximal site, the PPT was significantly lower at Days 3–7 (*P*<0.01 for all; Fig 3A) and no moderate or large effect sizes were observed. At the medial site, the PPT was significantly reduced respective to PRE on days 3–5 (*P*<0.01 for all; Fig 3B). Large (d = 0.840–0.990) and moderate (d = 0.730) effect sizes were noted for medial PPT at Days 3–4 and Day 7, respectively, with the PPT decrease from PRE being attenuated in the EPC group relative to the sham group. Finally, at the distal site, the PPT was significantly lower respective to PRE at Days 4–5 (*P*<0.01 for all; Fig 3C). For the distal PPT, large effect sizes were observed at Days 3, 4 and 7 (d = 0.970–1.160) and moderate effect sizes were observed at Days 5 and 6 (d = 0.720–0.740) again with the PPT decrease from PRE being attenuated in the EPC group respective to the sham group.

For flexibility measured via knee angle during the modified kneeling lunge, a main effect of time and group was observed, though there was no group*time interaction (Fig 3D). Knee angle at Days 3 and 5 was significantly greater than PRE measurements (*P*≤0.010) and a moderate effect size was observed at Days 4–7 (d = 0.660–0.770) such that the increase in knee angle from PRE in the EPC group was less than that observed in the sham group.

### Effects of EPC on lifting volume and isokinetic peak torque

There was a main effect of time (*P* = 0.031) for lifting volume over the 3 consecutive days of back squat exercise but no group (*P* = 0.834) or group*time interaction (*P* = 0.652). Lifting volume decreased from 5410 ± 524 kg to 5172 ± 571 kg to 5059 ± 518 kg in the EPC group for resistance training bouts 1–3, respectively, whereas lifting volume decreased from 5161 ± 383 kg to 5099 ± 412 kg to 4954 ± 445 kg in the sham group for resistance training bouts 1–3, respectively. However, lifting volume was not found to significantly decrease from resistance training bout 1 to bouts 2 or 3 (*P*>0.05 for all). No moderate or large effect sizes were observed at any time point for lifting volume.

Pre and post-testing measurements of isokinetic peak extensor torque (Days 1 & 7; i.e., PRE and POST2) at 60°/s did not demonstrate significant main effects or a group*time interaction (*P*>0.350 for all). Peak extensor torque decreased from 241.1 ± 13 N·m to 237 ± 17 N·m in the EPC group whereas it decreased from 235 ± 12 N·m to 226 ± 10.3 N·m in the sham group. No moderate or large effect size was observed for change in peak extensor torque.

### Effects of EPC on select blood markers of muscle damage and inflammation

**Fig 4:**
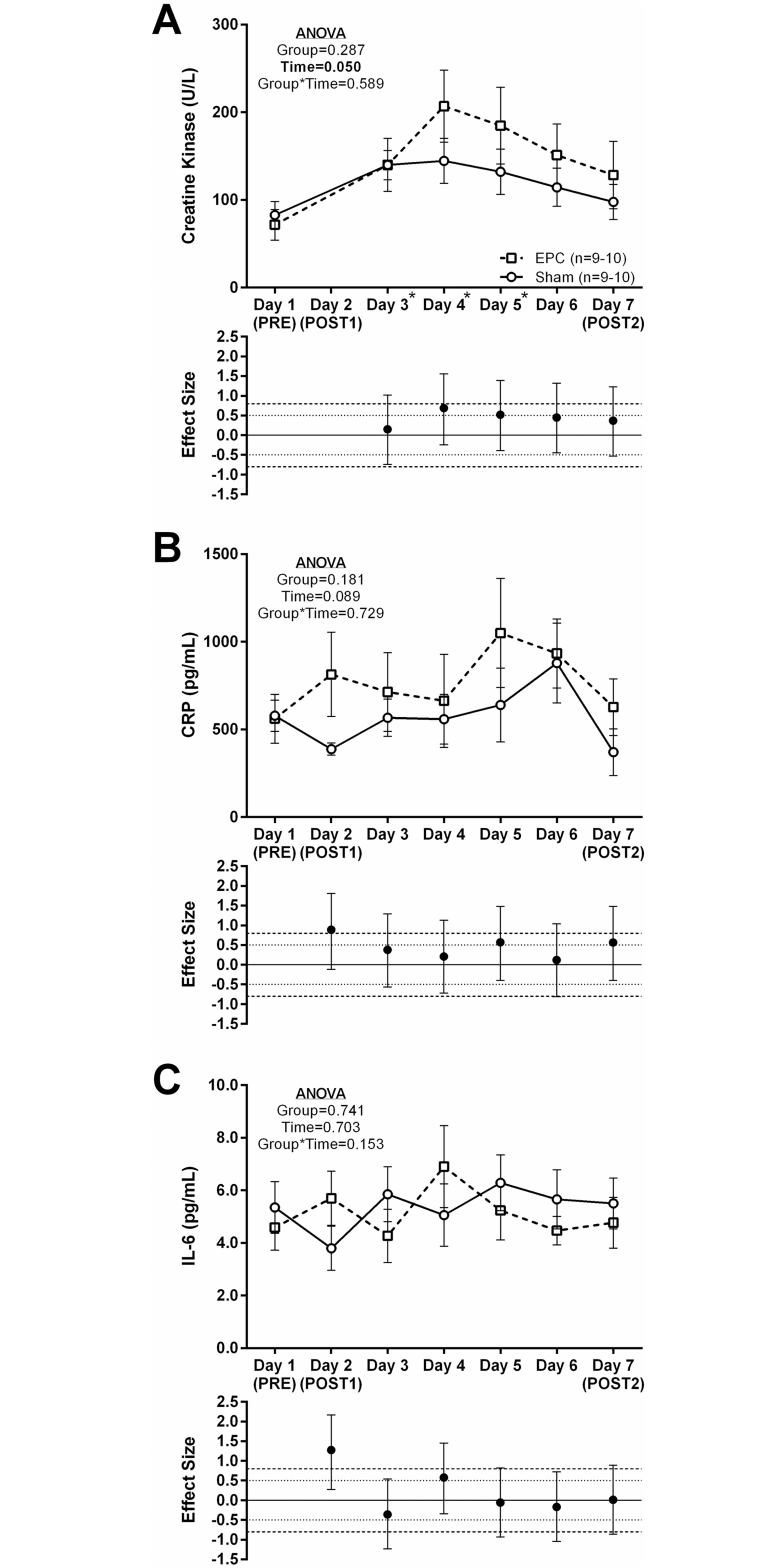
Humoral markers of muscle damage and inflammation. Creatine kinase (CK; Panel A), C-reactive protein (CRP; Panel B) and interleukin-6 (IL-6; Panel C) concentrations in the blood were measured at baseline (PRE/Day1) and on Days 3–7 of the protocol. Data are presented as mean respective concentrations ± standard error. Effect sizes were calculated as mean change from PRE in the EPC group less mean change from PRE in the sham group divided by the pooled standard deviation for each respective time point. Repeated measures MANOVA was performed with PRE values as a covariate and an α ≤ 0.05 required for statistical significance. Main effects of time and/or group are presented as emphasized p-values. *Post-hoc* testing for a main effect of time was performed using Student’s paired t-tests and an α ≤ 0.01 was required for statistical significance. *, time point (collapsed across groups) significantly different from PRE.

Serum CK activity demonstrated a main effect of time but no main effect of group or group*time interaction (Fig 4A). Across both groups, serum CK was significantly higher at Days 3–5 relative to PRE (*P*<0.001). In addition, moderate effect sizes were noted at days 3 and 4 (d = 0.523–0.688) during which there were higher concentrations of serum CK relative to PRE in the EPC group compared to the sham group.

Humoral concentrations of CRP did not demonstrate significant main effects or a group*time interaction (Fig 4B). However, a large (d = 0.887) and moderate (d = 0.566–0.570) effect size was noted at Days 2 and Days 5 and 7, respectively, with humoral CRP concentration change from PRE being larger in the EPC group relative to the sham group. Humoral concentrations of IL-6 also did not demonstrate significant main effects or a group*time interaction (Fig 4C) though a large (d = 1.27) and moderate (d = 0.577) effect size were noted at Days 2 and 4, respectively, with humoral IL-6 concentration change from PRE being larger in the EPC group relative to the sham group.

### Effects of EPC on muscle mRNAs related to hypertrophy and proteolysis

Targeted hypertrophy-related myokine and proteolysis-related mRNAs with EPC and sham are presented in Table 2. No significant main effects nor group*time interaction was observed for MGF, MSTN, or MuRF-1 mRNA. However, for MuRF-1 mRNA a large effect size (d = -1.083 [95% confidence interval: -1.99, -0.076]) was noted for change from PRE at the POST1 time point with a relative decrease occurring in the EPC group and a relative increase occurring in the sham group. A significant main effect of time, but no main effect of group or group*time interaction, was observed for atrogin-1 mRNA. However, neither the POST1 (*P* = 0.065) nor the POST2 (*P* = 0.123) time point were significantly different from PRE. Similar to MuRF-1 mRNA though, for atrogin-1 mRNA a moderate effect size (d = -0.516 [-1.41, -0.421]) was noted for change from PRE at the POST1 time point with a greater decrease occurring in the EPC group relative to the sham group.

**Table 2:** Effects of EPC during and following heavy resistance training on select PCR markers.

Marker(s)	Sham-POST1	Sham-POST2	EPC-POST1	EPC-POST2	ANOVA *P*-values		
					Group	Time	Group*Time
*Hypertrophy and proteolysis*							
MGF mRNA	1.23 ± 0.48	1.50 ± 0.53	1.32 ± 0.48	1.55 ± 0.36	0.908	0.220	0.983
MSTN mRNA	1.31 ± 0.71	2.35 ± 0.92	0.94 ± 0.14	1.51 ± 0.40	0.462	0.052	0.487
MuRF-1 mRNA	1.16 ± 0.21	1.29 ± 0.30	0.60 ± 0.09	1.05 ± 0.19	0.167	0.162	0.183
Atrogin-1 mRNA	0.92 ± 0.18	1.71 ± 0.53	0.70 ± 0.10	1.35 ± 0.39	0.471	**0.045**	0.655
*Ribosome biogenesis*							
c-Myc mRNA	27.5 ± 4.99	2.43 ± 0.77	31.5 ± 2.74	1.65 ± 0.39	0.591	<**0.001**	0.464
TAF1B mRNA	1.30 ± 0.25	2.24 ± 0.53	1.73 ± 0.46	3.37 ± 1.89	0.434	0.131	0.676
45s pre-rRNA	1.87 ± 0.48	1.85 ± 0.65	1.09 ± 0.15	1.30 ± 0.16	0.133	0.171	0.457
28s rRNA	1.27 ± 0.30	1.45 ± 0.47	1.07 ± 0.15	1.33 ± 0.10	0.663	0.148	0.882
Exportin-5 mRNA	1.48 ± 0.41	1.15 ± 0.31	1.07 ± 0.30	1.07 ± 0.30	0.585	0.452	0.617
*Inflammation*							
IL-6 mRNA	2.83 ± 1.02	1.86 ± 0.47	3.55 ± 1.29	1.40 ± 0.34	0.894	**0.018**	0.593
IL-10 mRNA	2.99 ± 1.10	1.82 ± 0.46	2.16 ± 0.65	1.09 ± 0.66	0.293	**0.047**	0.501
IL-1β mRNA	2.04 ± 0.51	0.75 ± 0.25	2.66 ± 0.52	0.64 ± 0.20	0.326	<**0.001**	0.188
MCP-1 mRNA	6.32 ± 1.94	1.93 ± 0.44	9.77 ± 2.40	2.30 ± 0.86	0.314	<**0.001**	0.292
TNF-α mRNA	3.32 ± 1.39	2.23 ± 0.78	2.50 ± 1.24	1.06 ± 0.49	0.437	0.064	0.679
*Satellite cell physiology*							
MyoD mRNA	3.98 ± 1.91	3.31 ± 2.38	3.03 ± 1.86	2.51 ± 1.11	0.675	0.182	0.934
Myogenin mRNA	1.99 ± 0.54	1.60 ± 0.25	1.57 ± 0.19	1.65 ± 0.33	0.655	**0.019**	0.646
Pax7 mRNA	5.86 ± 3.58	1.96 ± 0.86	2.87 ± 1.64	3.33 ± 2.16	0.747	0.165	0.444
MHCemb mRNA	1.93 ± 0.54	5.56 ± 1.81	1.77 ± 0.76	6.87 ± 3.98	0.801	**0.038**	0.793

All data are expressed as fold change (mean ± S.E.M., n = 8–10 subjects per target). Statistical comparisons from repeated measures ANOVA. Other notes: Abbreviations genes are in Table 1 which denotes primer sequences employed herein.

### Effects of EPC on muscle rRNAs and mRNAs related to ribosome biogenesis

Targeted mRNAs and rRNAs related to ribosome biogenesis with EPC and sham are presented in Table 2. A significant main effect of time, but no main effect of group or group*time interaction, was observed for c-Myc mRNA with expression significantly higher at the POST1 and POST2 time points relative to PRE (*P*<0.001). For 28S rRNA, 45S pre-rRNA, exportin-5 mRNA and TAF1B mRNA no significant main effects nor a group*time interaction was observed (*P*>0.05 for all). A moderate effect size (d = -0.746 [-1.64, -0.217] was noted for 45S pre-rRNA change from PRE at the POST1 time point such that the increase in expression was lesser in the EPC group relative to the sham group.

### Effects of EPC on muscle mRNAs related to inflammation

Targeted mRNAs related to inflammation with EPC and sham are presented in Table 2. A significant main effect of time was observed for IL-6, IL-10, IL-1β, and MCP-1 mRNA expression. IL-6 mRNA at the POST1 time point (*P* = 0.011), but not the POST2 time point (*P* = 0.043), was significantly elevated relative to PRE. Similarly, IL-10 mRNA was significantly elevated at the POST1 time point (*P* = 0.022) but not the POST2 time point (*P* = 0.240). However, a moderate effect size (d = -0.539 [-1.45, 0.426]) was observed at the POST2 time point such that the increase in IL-10 mRNA was reduced relative to that in the sham group. For IL-1β mRNA, expression was significantly greater relative to PRE at the POST1 (*P* = 0.002) but not POST2 (*P* = 0.066) time points. MCP-1 mRNA expression was also significantly greater relative to PRE at the POST1 (*P*<0.001), but not POST2 time point (*P*<0.030). For MCP-1 mRNA there was a moderate effect size (d = 0.501 [-0.41, 1.37]) observed at the POST1 time point at which the increase was greater in the EPC group relative to the sham group. Finally, no significant main effects nor a group*time interaction was observed for TNF-α mRNA expression.

### Effects of EPC on muscle mRNAs related to satellite cell physiology

Targeted mRNAs related to satellite cell physiology with EPC and sham are presented in Table 2. A significant main effect of time but no main effect of group or group*time interaction was observed for myogenin and MHCemb mRNA. For myogenin, mRNA expression levels at both the POST1 (*P* = 0.016) and POST2 (*P* = 0.005) time points was significantly higher relative to PRE. For MHCemb, mRNA expression levels at the POST2 (*P* = 0.005), but not the POST1 (*P* = 0.079), time point was significantly higher relative to PRE. Finally, no significant main effect of main effects or group*time interaction was observed for MyoD and Pax7 mRNA expression.

### Effects of EPC on protein expression patterns related to anabolic and proteolytic signaling

**Fig 5:**
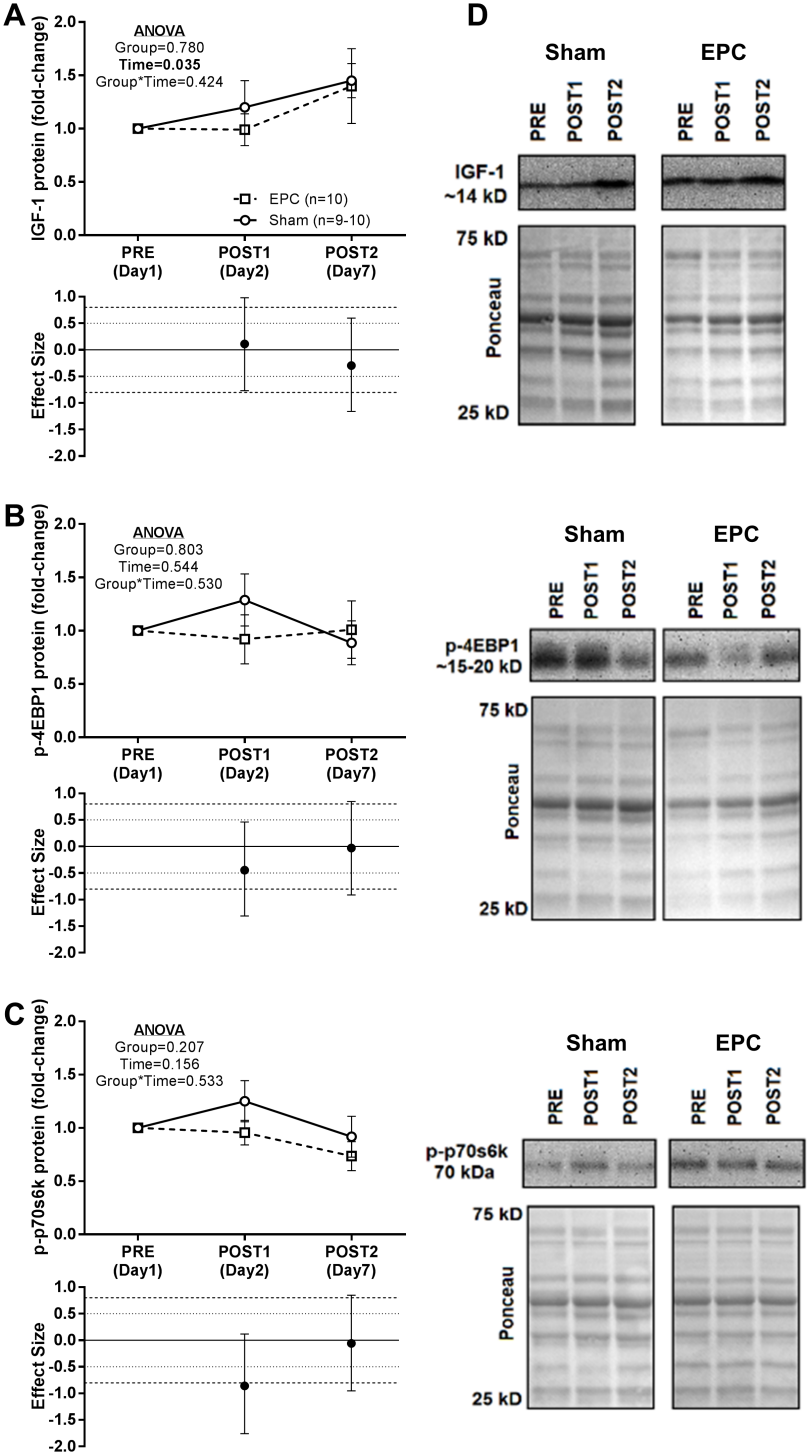
Protein expression patterns related to anabolic signaling. At baseline (PRE), 1 h following a heavy back squat resistance training session and treatment with EPC or sham (POST1) and 24 h following 3 consecutive days of heavy back squat resistance training session and treatment with EPC or sham and 2 additional, consecutive days of treatment with EPC or sham (POST2) protein expression patterns related to anabolic signaling were probed. Western blot analysis of protein concentrations in *vastus lateralis* biopsy samples are presented for A) insulin-like growth factor (IGF-1), B) phosphorylated 4E-binding protein 1 (p-4EBP1), and C) phosphorylated p-70 S6 kinase (p-p70s6k). Representative images and respective Ponceau images for all proteins are presented in Panel D. Protein expression data are expressed as fold-change from PRE levels (mean ± standard error) and effect sizes for change from PRE are presented as d ± 95% confidence interval. Effect sizes were calculated as mean change from PRE in the EPC group less mean change from PRE in the sham group divided by the pooled standard deviation for each respective time point. Repeated measures MANOVA was performed with PRE values as a covariate and an α ≤ 0.05 required for statistical significance. All statistics were performed using the absolute expression values. Main effects of time are presented as emphasized p-values. *Post-hoc* testing for a main effect of time was performed using Student’s paired t-tests and an α ≤ 0.025 was required for statistical significance.

Targeted protein expression patterns related to anabolic and proteolytic signaling with EPC and sham are presented in Fig 5. No significant main effect of group or group*time interaction was observed for IGF-1, p-4EBP1 or p-p70s6k. A significant main effect of time was observed for IGF-1, but not p-4EBP1 and p-p70s6k (Fig 5A–5C). However, p*ost-hoc* analysis did not reveal any significant differences from PRE at the POST1 (*P* = 0.7128) or POST2 (*P* = 0.052) time points for IGF-1 protein expression. A large effect size (d = -0.857) for IGF-1 was noted at the POST1 time point as there was an increase in the sham group and a decrease in the EPC group relative to PRE.

**Fig 6:**
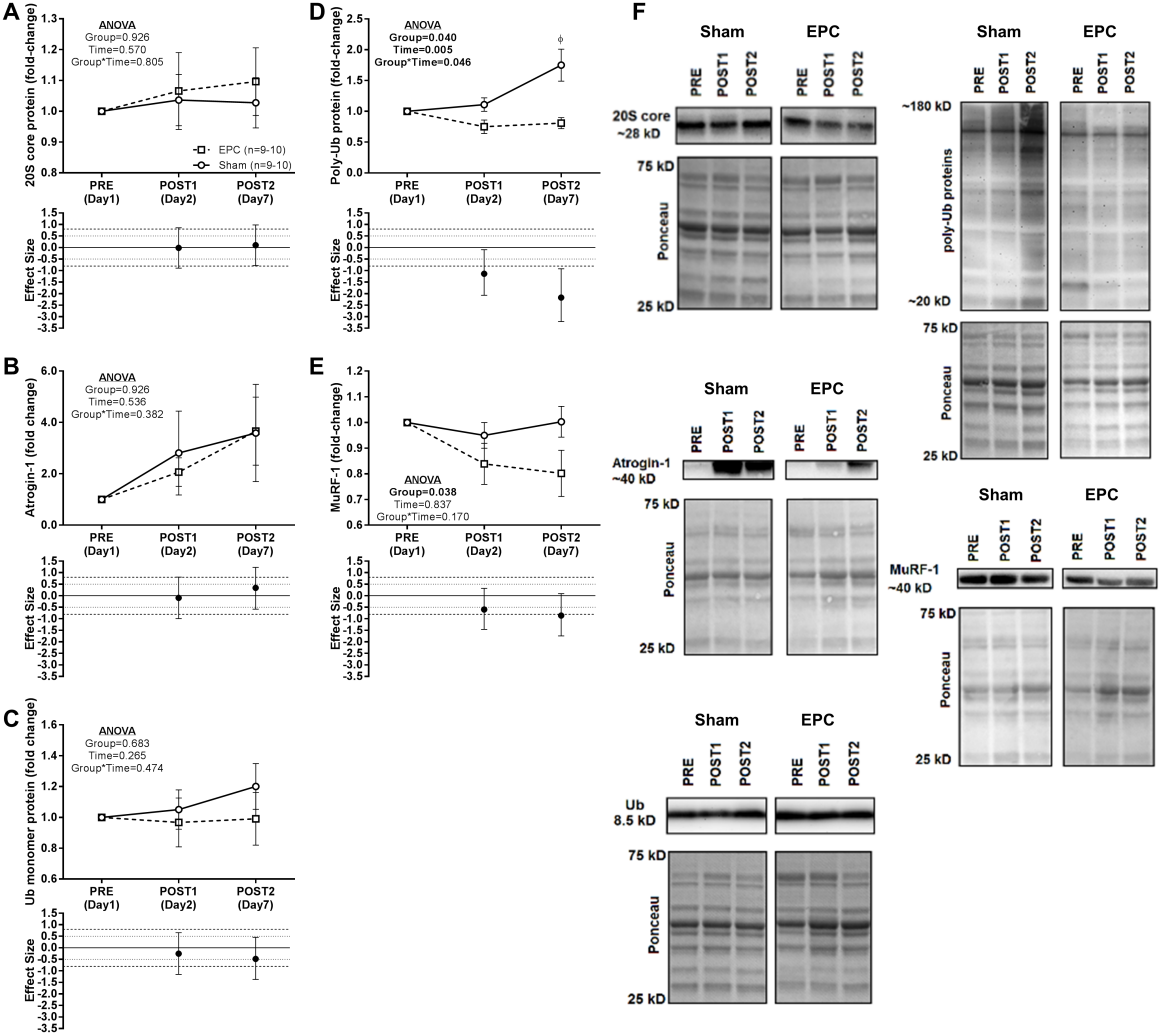
Protein expression patterns related to proteolytic signaling. At baseline (PRE), 1 h following a heavy back squat resistance training session and treatment with EPC or sham (POST1) and 24 h following 3 consecutive days of heavy back squat resistance training session and treatment with EPC or sham and 2 additional, consecutive days of treatment with EPC or sham (POST2) protein expression patterns related to proteolytic signaling were probed. Western blot analysis of protein concentrations in *vastus lateralis* biopsy samples are presented in panels A) 20S core proteasome protein, B) atrogin-1, C) Ubiquitin (Ub) monomer protein, D) poly-ubiqutinated (poly-Ub) proteins, and E) MuRF-1. Representative images and respective Ponceau images for all proteins are presented in Panel F. Protein expression data are expressed as fold-change from PRE levels (mean ± standard error) and effect sizes for change from PRE are presented as d ± 95% confidence interval. Effect sizes were calculated as mean change from PRE in the EPC group less mean change from PRE in the sham group divided by the pooled standard deviation for each respective time point. Repeated measures MANOVA was performed with PRE values as a covariate and an α ≤ 0.05 required for statistical significance. All statistics were performed using the absolute expression values. Main effects and/or group*time interactions are presented as emphasized p-values. *Post-hoc* testing for between time point differences (independent of group) and between group differences at the POST1 and POST2 time points were performed using Student’s paired t-tests and an α ≤ 0.025 was required for statistical significance. ϕ, significantly different from sham at same time point.

No main effects nor a group*time interaction was observed for 20S proteasome core, atrogin-1, or Ub-monomer protein expression (Fig 6A–6C). However, for poly-Ub proteins, significant main effects of time and group and a significant group*time interaction were observed (Fig 6D). Across groups, poly-Ub was not significantly higher at the POST1 (*P* = 0.181) or POST2 (*P* = 0.266) time points relative to PRE though poly-Ub was significantly higher in the sham group compared to the EPC group at the POST2 time point (*P* = 0.001). Moreover, large effect sizes were observed at the POST1 (d = -1.14) and POST2 (d = -2.17) time points such that there were decreases in the EPC group relative to increases in the sham group. Finally, for MuRF-1 a significant main effect of group, but no main effect of time or group*time interaction, was observed (Fig 6E). A moderate (d = -0.595) and large (d = -0.860) effect size was observed at the POST1 and POST2 time points, respectively, with a greater decrease in MuRF-1 protein expression relative to PRE in the EPC group compared to the sham group.

### Effects of EPC on protein expression patterns related to inflammatory signaling and oxidative stress

**Fig 7:**
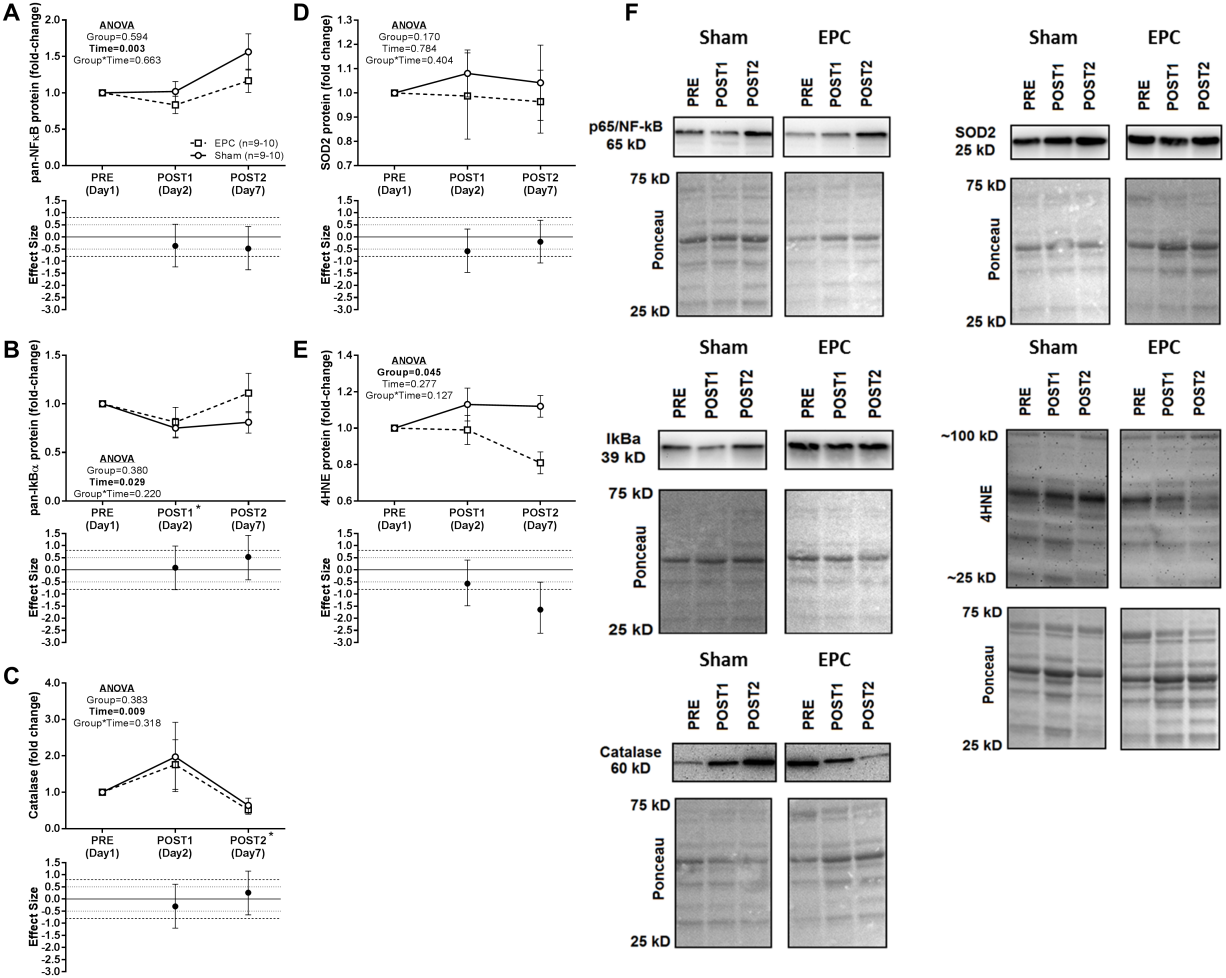
Protein expression patterns related to inflammatory and oxidative stress signaling. At baseline (PRE), 1 h following a heavy back squat resistance training session and treatment with EPC or sham (POST1) and 24 h following 3 consecutive days of heavy back squat resistance training session and treatment with EPC or sham and 2 additional, consecutive days of treatment with EPC or sham (POST2). Western blot analysis of protein concentrations in *vastus lateralis* biopsy samples are presented for A) pan-nuclear factor kappa-light change-enhancer of activated B cells (pan-NF-κB), B) pan-nuclear factor of kappa light polypeptide gene enhancer in B-cells inhibitor, alpha (pan-IκBα), C) catalase, D) superoxide dismutase 2 (SOD2), and E) 4-hydroxynonenal (4HNE). Representative images and respective Ponceau images for all proteins are presented in Panel F. Protein expression data are expressed as fold-change from PRE levels (mean ± standard error) and effect sizes for change from PRE are presented as d ± 95% confidence interval. Effect sizes were calculated as mean change from PRE in the EPC group less mean change from PRE in the sham group divided by the pooled standard deviation for each respective time point. Repeated measures MANOVA was performed with PRE values as a covariate and an α ≤ 0.05 required for statistical significance. All statistics were performed using the absolute expression values. Main effects are presented as emphasized p-values. *Post-hoc* testing for between time point differences (independent of group) were performed using Student’s paired t-tests and an α ≤ 0.025 was required for statistical significance. *, time point (collapsed across groups) significantly different from PRE.

Protein expression patterns related to inflammatory signaling and oxidative stress with EPC and sham are presented in Fig 7A–7E. A main effect of time was observed for both pan-NF-κB (Fig 7A) and pan-IkBα (Fig 7B) without a main effect of group or group*time interaction. For NF-κB., protein expression across groups was not significantly different from PRE at either the POST1 (*P* = 0.115) or POST2 (*P* = 0.040) time points. IkBα protein expression across groups was significantly lower than PRE at the POST1 time point (*P* = 0.006) but not different from PRE at the POST2 time point (*P* = 0.311). However, for pan-IkBα a moderate effect size (d = 0.532) was observed at the POST2 time point as there was relatively little change from PRE in the EPC group compared to a decrease in the sham group.

For catalase, a main effect of time but no main effect of group or group*time interaction was observed (Fig 7C). *Post-hoc* analysis revealed a statistically significant change in catalase protein expression relative to PRE at the POST2 (*P* = 0.005) but not the POST1 (*P* = 0.356) time point. No main effects or group*time interaction were observed for SOD2 protein expression (Fig 7D) though a moderate effect size (d = -0.586) was noted at the POST1 time point with an increase from PRE in the sham group and a decrease from PRE in the EPC group. For 4HNE, no main effect of time or group*time interaction was observed, but there was a significant main effect of group (Fig 7E). Moreover, a moderate (d = -0.586) and large (d = -1.64) effect size for change from PRE was observed at the POST1 and POST2 time points, respectively, with lower 4HNE levels in the EPC group compared to sham.

## Discussion

Given our initial research questions and ensuing investigation seeking to elucidate differences between EPC and sham groups in select molecular and performance-related variables in the context of intense, voluminous resistance exercise, we consider the following main findings from this experiment: 1) the barbell back squat sessions employed herein sufficiently disrupted homeostasis as demonstrated by significant changes (compared to baseline data) in select performance-related and molecular variables over the course of the study period in both groups. These changes included decreases in flexibility and the PPT, increases in blood markers of muscle damage and inflammation, alterations in muscle mRNA expression patterns related to proteolysis, ribosome biogenesis, inflammation, and markers of satellite cell activity, and alterations in protein expression patterns related to anabolic and proteolytic signaling, inflammatory signaling and oxidative stress. 2) There were several moderate-to-large effects of EPC relative to sham at various time points including, but not limited to, flexibility and PPT and MuRF-1, poly-ubiquitinated (poly-Ub) and 4HNE protein expression.

Although controversial data exists regarding causal relationships between a lack of flexibility or “muscle tightness” and injury/performance, some investigations indicate muscle tightness, as a relatively inherent by-product of training and competition, places individuals at a greater risk of injury [20, 21]. To our knowledge, we are the first to investigate the effects of EPC on flexibility over the course of several days in conjunction with heavy, voluminous resistance exercise. Other investigators have demonstrated that EPC acutely enhances flexibility, specifically in a forward split [8]. Importantly, in that study flexibility was only assessed immediately before and after a single EPC treatment independent of any exercise induced muscle damage whereas we assessed flexibility ~22–23 h following each EPC treatment bout (and resistance training when applicable). We found that flexibility about the knee joint during a modified forward lunge was significantly lower in the sham group across Days 2–7 (i.e. main effect of group) and that there were moderate effect sizes on Days 4–7 indicating an attenuation in the increase in knee angle with EPC treatment. Since this resistance exercise intervention was only sub-chronic, the differences in flexibility are not likely explained by significant changes in tissue length. Mechanistically, as proposed by Sands and colleagues [8], these findings may be related to the thixotropic properties of muscle tissue. This property of muscle is related to the sarcoplasm shifting from a “gel-like” state, which would be more resistant to lengthening, to a more “liquid-like” state that would be less resistant to lengthening [8]. Given the nature of this EPC treatment, the compression and therefore perturbation of the sarcoplasm of muscle cells could hypothetically reduce the viscosity of the medium through which the sarcomere shortens and lengthens augmenting flexibility.

The impaired flexibility in sham may also be related to residual cross-bridge attachment resisting stretch, particularly observed in sore muscle [8, 22, 23]. Indeed, other investigators have reported significant reductions in joint range of motion when subjects presented with muscle soreness [24, 25] which PPT functioned as a proxy of in this investigation. We observed moderate-to-large effect sizes on the PPT on 3 occasions (Days 3, 4 and 7; 60% of non-PRE measurement days) and 5 occasions (Days 3–7; 100% of non-PRE measurement days) at the medial and distal sites, respectively, indicating an EPC-mediated attenuation of soreness. These findings would appear to be in agreement with Sands and colleagues who reported that a single 15 min EPC treatment was associated with significantly higher PPT (i.e. lower soreness) immediately following treatment and after a subsequent bout of sport training, though these measurements were all performed in the same day [9]. Algometry has been widely employed as a more objective means by which the extent of muscle soreness can be quantified and has also proven to be a reliable, valid method so long as consistent, standard operating procedures are implemented [9, 26–28]. However, although PPT measurements via algometry functioned as a proxy of muscle soreness in this investigation, neither thixotropic-related changes in pertinent musculature nor residual cross-bridge attachment resisting stretch were directly measured and their role in the observed responses should be investigated further. Regardless, these findings can be simply explained as reductions in *vastus lateralis* muscle soreness via EPC usage when compared to passive recovery/sham alone. Interestingly, soreness did not appear to be differentially affected at the proximal site which we posit could be due to the coverage (or lack thereof) of the compression sleeves.

Aside from the more applied scientific findings related to flexibility and soreness differences between groups in this investigation, differences between groups also existed for markers of muscle damage, inflammation, proteolytic signaling, and oxidative stress. Specifically, the following findings will be briefly discussed below: 1) increases in serum CK and inflammatory marker levels were more robust at some time points, particularly on Days 2 and/or 3, 2) moderate-to-large effect sizes on atrogin-1 and MuRF-1 mRNA expression at the POST1 time point relative to PRE at which expression was reduced in the EPC group, 3) significantly higher muscle poly-Ub protein concentrations at the POST2 time point in the sham group compared to EPC and significantly higher MuRF-1 protein levels across the POST1 and POST2 time points in the sham group compared to the EPC group, and 4) significantly lower 4HNE levels across the POST1 and POST2 time points in the EPC group compared to sham.

A number of others have shown significant increases in serum CK levels following damage to skeletal muscle cells at the sarcolemmal and z-disk level [29] and CK functioned herein as a gross marker of muscle damage. We observed relatively similar serum CK levels between groups though concentrations were non-significantly more pronounced at some time points in the EPC group. A similar pattern was noted with CRP concentrations in the EPC group and we posit that the compressive stimulus may “mobilize” a greater proportion of metabolites/cellular contents in the context of sarcolemmal injury to the extracellular space. However, Cochrane and colleagues have reported similarity in CK levels when comparing an EPC subject pool to a non-treatment control group after strenuous eccentric exercise [30]. Given the quantitative and qualitative similarity in this metric, other investigators findings, and the influence of other confounding variables when analyzing serum CK levels (i.e. genotype, training age, time of day, etc.), this finding warrants further targeted research [31] though clearly demonstrates the resistance training protocol employed was sufficient to cause marked skeletal muscle damage.

While muscle protein synthesis (MPS) and muscle protein breakdown (MPB) rates were not directly measured herein, select molecular targets related to each process were employed to compare any differential responses between groups. Although our elected mRNA and protein targets related to canonical MPS pathways revealed no significant differences between groups (i.e. MGF, IGF-1, p-4EBP1, p-p70s6k), differential effects of EPC and sham on markers of MPB emerged. Each of the MBS-related mRNA and protein targets we examined are components of the ubiquitin proteasome system (UPS). Briefly, in muscle tissue, the UPS is a proteolytic system that degrades target proteins that have been poly-ubiquinated with multiple ubiquitin monomers through the coordinated process of three enzymes (“E1”, “E2”, and “E3”), [32, 33]. Numerous investigators have examined constituents of this system in the context of resistance exercise seeking to offer explanatory mechanisms for the increase in muscle protein breakdown (MPB) observed after bouts of resistance exercise [34–37] and we refer the reader there for a more comprehensive description.

Atrogin-1 and MuRF-1 are two, muscle-specific E3 ligases catalyzing the conjugation of ubiquitin to a substrate protein and have been shown to be specific to contractile proteins [33, 38]. The 20S core protein is the “catalytic” portion of the 26S proteasome and is involved in the degradation of contractile proteins [39]. Herein we did note moderate-to-large effect sizes for atrogin-1 and MuRF-1 mRNA expression at the POST1 time point suggesting a blunted or decreased response with EPC treatment compared to sham. In addition, there was a significant group (i.e. EPC) effect on poly-Ub and MuRF-1 protein expression with a large effect noted at the POST2 time point, which was approximately 72 hours after the final bout of resistance exercise. Others have shown similar increases in MuRF-1 mRNA, ubiquitin protein, and poly-Ub proteins up to 48h after resistance exercise, but to our knowledge expression of these targets at a time point corresponding to 72 hours after a bout or successive bouts of resistance exercise has not been reported which limits our ability to compare our findings [34, 40]. However, considering our proteolytic signaling data holistically, EPC appears to down-regulate proteolytic signaling when used concurrently with sub-chronic resistance training. We are unaware of any evidence revealing a concise mechanism whereby EPC may down-regulate proteolytic signaling in muscle tissue. In fact, in contrast to our findings, Siu and colleagues reported an immediate, significant increase in atrogin-1 protein expression following 6 h of static compression at ~100 mmHg in rats [41] suggesting that the dynamic nature (or lack thereof) of the compression stimulus may be critical to the proteolytic signaling response. Thus, more research delineating these aspects of the recovery-adaptation response to resistance training when employing EPC is warranted.

4-hydroxynonenal (4HNE) is a resultant protein by-product of the lipid peroxidation of ω6-polyunsaturated fatty acids under conditions of oxidative stress and functioned as a proxy of oxidative stress in this study [42, 43]. Local oxidative stress seems to have been significantly reduced by EPC in this protocol as evidenced by the significant decrease in 4HNE levels observed in the EPC group across the POST1 and POST2 time points (Fig 7E). The significant decline in 4HNE levels observed in the EPC group may indicate EPC-mediated oxidative stress resilience in conjunction with resistance exercise. Interestingly, in an earlier investigation examining the effects of a single EPC treatment without exercise we reported that: 1) SOD2 mRNA levels tended to increase 1 h post-treatment, and 2) SOD2 protein levels tended to increase 4 h post-treatment; both of these findings being suggestive that EPC may upregulate SOD2 which, in turn, increases oxidative stress resilience [10]. However, in another investigation employing 1 week of EPC treatment without exercise we reported that 7 consecutive days of EPC treatment increases 4HNE levels in skeletal muscle without affecting SOD2 or catalase protein levels [12]. However, in the latter investigation, the increase in 4HNE was associated with a lower target inflation pressure (~30mmHg) compared to that utilized herein (~70 mmHg). In our current EPC/exercise model, SOD2 or catalase protein levels were unaltered despite decrements in 4HNE levels in the EPC group. While our previous findings are difficult to reconcile with our current findings, it remains possible that EPC induces oxidative stress resilience independent of SOD2 and/or catalase gene regulation. In this regard, we have reported that 7 days of EPC upregulates sulfridoxin-1 mRNA which encodes for a protein that plays a role in oxidative stress protection [12]. Hence, our data collectively suggests the following: 1) EPC treatments without exercise seemingly upregulates oxidative stress which, in turn, increases the expression of genes related to oxidative stress resilience and 2) EPC with resistance exercise prevents summative increases in oxidative stress and this may be related to the aforementioned upregulation in genes (aside from SOD2 and catalase) that promote oxidative stress resilience while also potentially lowering prolonged muscle proteolysis. Importantly, the current study is a third line of evidence suggesting that there is interplay between EPC and oxidative stress and more mechanistic research is needed in order to continue exploring this relationship.

In conclusion, this is one of the first studies to our knowledge which has used EPC as an adjuvant to sub-chronic resistance training with the intent of characterizing functional and molecular recovery-related responses. Our data suggest that EPC reduces muscle soreness and attenuates reductions in flexibility, albeit the mechanisms through which this occurs need to be further elucidated. Likewise, the potential EPC-induced reduction in muscle proteolysis as well as oxidative stress warrant further mechanistic research.
